# Putting rapid tests to work in surveillance and control of cholera

**DOI:** 10.1016/j.cmi.2021.10.012

**Published:** 2021-10-29

**Authors:** Andrew S. Azman, Iza Ciglenecki, Francisco J. Luquero

**Affiliations:** 1) Department of Epidemiology, Johns Hopkins Bloomberg School of Public Health, Baltimore, MD, USA; 2) Médecins sans Frontières, Geneva, Switzerland; 3) Institute of Global Health, Faculty of Medicine, University of Geneva, Geneva, Switzerland; 4) Gavi, the Vaccine Alliance, Geneva, Switzerland; 5) Department of International Health, Johns Hopkins Bloomberg School of Public Health, Baltimore, MD, USA

The Global Taskforce for Cholera Control (GTFCC) End Cholera Roadmap encourages countries with endemic and epidemic cholera transmission to identify cholera hotspots in order to prioritize multi-sectoral prevention and control strategies, including oral cholera vaccines and water/sanitation infrastructure [1]. Although cholera is inextricably linked to poverty, including limited access to safe water and sanitation, there is limited predictive value in common metrics related to these for identifying where cholera incidence or mortality risk is high. The primary, though imperfect, predictor we have for identifying high-risk cholera areas is historical cholera incidence. However, laboratory diagnosis of cholera is extremely uncommon. What many call ‘cholera’ incidence is typically just the incidence of suspected cholera (for example, ref. [2]), which during a declared outbreak is any person with acute watery diarrhoea. Often more than half of these cases are caused by other enteric pathogens, not *Vibrio cholerae*, and this proportion probably varies by location and over time [3]. In other settings, true cholera cases are not reported, even as suspected cases, because they have not been laboratory confirmed.

Current World Health Organization (WHO) recommendations suggest that laboratory confirmation should be performed to confirm the start and end of outbreaks, and that ‘it may be interesting to take a few samples randomly during the outbreak to make sure that the antimicrobial sensitivity pattern of the pathogen has not changed’ [4]. Unfortunately, the classic reference standard confirmation method, culture, is not routinely performed and capacity to perform cholera culture is often limited to a single national reference laboratory in many cholera-affected countries. Culture can suffer from moderate false-negative rates (15%–30% [5,6]), which can be exacerbated by poor sample handling, inadequate transportation, lack of adherence to recommended standard operating procedures and previous antibiotic use—a common reality in cholera endemic areas. PCR is more sensitive but is rarely available—even at national reference laboratories—and there is no widely agreed upon PCR protocol for toxigenic *V. cholerae* O1 (the strain most likely to cause outbreaks and distinct from native environmental Vibrios that may cause sporadic diarrhoea).

Fortunately, a handful of immunochromatographic lateral-flow assays, referred to as rapid diagnostic tests (RDTs), are available on the market today. In this issue of *Clinical Microbiology and Infection*, Muzembo et al. conduct a systematic review and meta-analysis of the evidence accumulated over more than 15 years on the performance of these tests [7]. The meta-analysis reveals that these tests, when used directly on stool have a sensitivity of 91% (95% CI 87%–94%) and a specificity of 80% (95% CI 74%–84%) compared with culture or PCR. After enriching samples in a selective growth medium for multiple hours (often 4–6 hours), there appears to be a moderate gain in specificity (98%; 95% CI 95%–99%) with almost no change in sensitivity. Although this meta-analysis has a number of shortcomings, including the pooling of many different tests (different chemistry, changes over time of the same test), differences in testing protocol adherence and sampling conditions, and use of an imperfect reference standard, they provide a much needed synthesis of the evidence to date and help to highlight the practical utility and limitations of these tests.

If used appropriately, RDTs can help us transition from making epidemiological inference and public health decisions on counts of acute watery diarrhoea cases to counts of acute water diarrhoea caused by *V. cholerae*. Recent use of RDTs in both endemic and outbreak situations, including Yemen where more than 400 000 RDTs (roughly 25% of the suspected cases reported with a 40% positivity rate) have been used since 2018, make it clear that decentralized RDT use can be possible even in some of the most challenging field conditions (Yemen EOC Dashboard, accessed 21 September 2021). Although broad use of RDTs is possible, a number of critical policy and scientific steps are needed to move towards rational widespread use of these diagnostic tools.

## RDT performance standards and quality assurance

Rigorous standards for cholera RDTs in terms of both performance and quality control are needed to ensure that only high-quality tests are used and to allow for appropriate interpretation of results at the individual and population-levels. Over the past decade, manufacturers of commonly used RDTs have made significant and often undocumented changes to their devices without appropriate communication to end users, eroding trust and making it hard to compare results of validation studies conducted in different time periods [8]. In 2017, the GTFCC convened a group of experts to develop a target product profile, which later led to the development of technical specifications for the WHO-prequalification of cholera RDTs [9,10]. Based on the pooled analyses in Muzembo et al., it is unclear whether the specificity of any RDTs used directly on stool will meet the target product profile, although test-specific pooled analyses restricted to only the current generation of tests may prove otherwise. At the time of writing, there has yet to be a single cholera RDT prequalified. More work is needed to encourage manufacturers to submit their dossiers for prequalification and to ensure rapid review by the WHO prequalification office.

## Improvements in surveillance guidance and appropriate use

Although new guidance from WHO on RDT use has not been released, interim guidance on RDT use from the WHO-led GTFCC in 2015 stated that an RDT positive is sufficient to launch a cholera alert but not a confirmed outbreak, which requires culture or PCR results [11]. This interim guidance also states that RDTs can rule out cholera if all are negative and that they are of limited clinical utility. Unfortunately, there is no clear guidance for ministries of health on how to use these tests as part of their larger surveillance system. In areas where there is no known cholera transmission, decentralized placement of RDTs can indeed help to rapidly identify outbreaks, allowing authorities to respond more quickly. In areas where cholera cases are regularly reported, or during an outbreak, regular and decentralized use of RDTs can help us understand the true incidence of medically attended cholera. The clinical utility of cholera RDTs is less obvious, but it is not negligible and further exploration of this use-case may help with improved systematic use by clinicians. The imperfect specificity of RDTs can lead to false positives; however, a positive test in a patient with acute watery diarrhoea significantly increases the post-test probability of it being a real cholera case. By knowing this, clinicians might better prioritize who to target for antibiotics (e.g. among mild cases, aiming to reduce shedding post-discharge) and which antibiotics are likely to be useful.

One-size-fits-all recommendations will be challenging, if not impossible, to develop, but we urge simple guidance that clinical staff can implement and understand. For example, testing all of the first five suspected cases each day at each facility then sampling every tenth suspected case after that. This type of guidance can help ministries of health to plan for the procurement of RDTs, integrate RDT results into routine surveillance system reports (e.g. District Health Information Software) and allow for a more refined picture of cholera locally and globally. Although it appears that testing of samples after a multi-hour enrichment step improves specificity, this comes with operational shortcomings (i.e. it makes the rapid test no longer rapid) and guidance on when to use tests directly on stool or after enrichment, perhaps depending on context (e.g. in district laboratories rather than in the cholera treatment units), would further improve adoption of RDTs.

## Further research on RDTs

Our understanding of the current generation of RDTs is far from perfect. Almost all field evaluations of RDTs have reported false-positive results, but this has not been well replicated in laboratory studies [12]. Making evidence-based guidance on the number of tests to perform often requires assumptions about the independence of test results and with better understanding of the false-positive mechanisms (e.g. related to test batch, specific *V. cholerae* strain, co-circulating pathogen, ‘random chance’, or simply an insensitive reference standard) more appropriate guidance can be crafted, especially that relating to the detection of outbreaks. As shown by Muzembo et al., the heterogeneity of performance estimates across studies has been extremely large across settings and tests. A well-controlled head-to-head field comparison of quality-assured RDTs against both PCR and culture would greatly improve our ability to understand the real differences between assays and their actual field performance when conditions are close-to-ideal.

Looking to the future, the current generation of commercially available RDTs may not be sufficient. As countries progress on their pathway to cholera elimination, the specificity of the current generation of tests (up to 90%) may be too low. This includes both the analytical specificity in detecting *V. cholerae* O1 and the fact that in many places even after cholera transmission is largely eliminated, we may expect to see diarrhoea caused by non-toxigenic *V. cholerae* O1, which are not typically involved in large epidemics but will test positive with current RDTs [13]. New rapid tests, whether immuno-based or molecular, are needed to further reduce false-positive results. Ideally, the new generation of tests should help clinicians to decide on appropriate antibiotic use in a context of growing concern of antimicrobial resistance. Furthermore, as we rely more on genomics for the classification of circulating *V. cholerae* strains, more work is needed to understand whether the RDTs used could serve as an appropriate medium to temporarily preserve and transport the genetic material needed for subsequent molecular analyses, as has been done with filter paper [14].

Culture and PCR both have critical roles to play in the cholera diagnostics landscape, but RDTs provide an important avenue to decentralize (e.g. expand testing to more primary-care facilities) and refine surveillance systems. These simple tools, while imperfect, can sharpen our view of this disease by distinguishing true cholera from other causes of acute watery diarrhoea. This sharpened view will help the global community more efficiently target cholera resources, including vaccines, and make progress towards the goal of ending cholera as a public health threat by 2030 and beyond.
